# T2* mapping in an equine articular groove model: Visualizing changes in collagen orientation

**DOI:** 10.1002/jor.24764

**Published:** 2020-06-10

**Authors:** Sander Brinkhof, Nikae te Moller, Martijn Froeling, Harold Brommer, René van Weeren, Keita Ito, Dennis Klomp

**Affiliations:** ^1^ Department of Radiology University Medical Center Utrecht Utrecht The Netherlands; ^2^ Department of Clinical Sciences, Faculty of Veterinary Medicine Utrecht University Utrecht The Netherlands; ^3^ Department of Orthopaedics University Medical Center Utrecht Utrecht The Netherlands; ^4^ Department of Biomedical Engineering, Orthopaedic Biomechanics Eindhoven University of Technology Eindhoven The Netherlands

**Keywords:** cartilage, groove model, MRI, T2* mapping

## Abstract

T2* mapping is promising for the evaluation of articular cartilage collagen. In this work, a groove model in a large animal is used as a model for posttraumatic arthritis. We hypothesized that T2* mapping could be employed to differentiate between healthy and (subtly) damaged cartilage. Eight carpal joints were obtained from four adult Shetland ponies that had been included in the groove study. In this model, grooves were surgically created on the proximal articular surface of the intermediate carpal bone (radiocarpal joint) and the radial facet of the third carpal bone (middle carpal joint) by either coarse disruption or sharp incision. After 9 months, T2* mapping of the entire carpal joint was carried out on a 7.0‐T whole‐body magnetic resonance imaging (MRI) scanner by means of a gradient echo multi‐echo sequence. Afterwards, assessment of collagen orientation was carried out based on Picrosirius Red‐stained histological sections, visualized by polarized light microscopy (PLM). The average T2* relaxation time in grooved samples was lower than in contralateral control sites. Opposite to the grooved areas, the “kissing sites” had a higher average T2* relaxation time than the grooved sites. PLM showed mild changes in orientation of the collagen fibers, particularly around blunt grooves. This work shows that T2* relaxation times are different in healthy cartilage vs (early) damaged cartilage, as induced by the equine groove model. Additionally, the average T2* relaxation times are different in kissing lesions vs the grooved sites.

## Graphical abstract

The median T2* relaxation time was lower in the sample group of grooved cartilage than in contralateral control sites. The sample group of kissing sites had a higher median T2* relaxation time as compared to the opposite, grooved sites. This work shows that T2* relaxation times are different in healthy cartilage vs (early) damaged cartilage, as induced by the equine groove model.

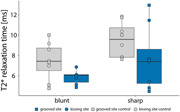

## 1 INTRODUCTION

Osteoarthritis (OA) is a progressive whole‐joint disease which is characterized by degeneration of the articular cartilage, synovitis, and bone remodeling in synovial joints resulting in patients suffering from joint stiffness reduced range of motion and often pain.^1^ This cartilage degeneration is characterized by loss of glycosaminoglycans (GAGs) and alterations to the collagen fiber network.^2^ Although, up until today, there is no cure for osteoarthritis, early detection of cartilage damage is essential for (future) therapy planning in order to prevent or decelerate the progressive irreversible damage to the joint.

T2* mapping is sensitive to water content and to the degree of orientation of the collagen fiber network within the articular cartilage.^3^ In addition, zonal variations in the depth of the articular cartilage could be visualized by employing T2* mapping.^4^ Since articular cartilage has a high curvature, T2* mapping is ideally implemented on an ultra‐high field scanner to achieve a high spatial resolution which is needed to correctly map the thin layer of cartilage. It has been shown that T2* mapping is feasible on a 7T magnetic resonance imaging (MRI) with diagnostic imaging quality.^5^ However, T2* relaxation time on 7T is significantly shorter than on 3T, which has to be taken into account for the imaging protocols used.^6^ T2* mapping has been successfully applied within a number of joints in the human body, such as the hip,^5^, ^7^ knee,^8^, ^9^ and ankle.^10^, ^11^ To test whether T2* mapping could serve as a biomarker for (early) cartilage degeneration in a controlled fashion, one could employ an animal model such as a groove model, serving as a model for posttraumatic arthritis.

The cartilage groove model has been used for OA research in rats,^12^ dogs,^13^ sheep,^14^ and horses,^15^ and has shown its potential to create posttraumatic arthritic changes in the joint. In this model, the cartilage layer is grooved through the superficial, middle, and deep zone (leaving the calcified layer intact) and the joint is subjected to intensified loading. The horse in particular is an interesting model because of the similarities between equine and human cartilage.^16^ Recently, the groove model has been applied in the equine carpal joint in two variants: grooves were surgically created by either blunt disruptions or sharp incisions (blunt grooves and sharp grooves, respectively). The contralateral joint was sham‐operated and used as a control.^17^

It is believed that blunt grooves and sharp grooves create different types of damage and, consequently, different progression of the disease. In this study, we investigated the difference in cartilage integrity between grooved and control sites and between the two groove types. For that purpose, ultra‐high field MRI was employed by means of a T2* mapping sequence to gain insight into cartilage quality. T2* is sensitive to magnetic susceptibility differences, and shortens in proximity to transitions between tissues like water to bone. We hypothesized that T2* relaxation times (a) are shorter in grooved cartilage than in control cartilage, (b) are different in the “kissing sites” (ie, the contact surface of the grooved cartilage) compared with grooved cartilage or control cartilage, and (c) can distinguish between blunt and sharp grooves.

## 2 METHODS

### 2.1 Subjects

Four adult female Shetland ponies with a mean age of 7.3 years (SD, 3.9 years; range, 4‐13 years) and with a mean body weight of 203 kg (SD, 21.8 kg; range, 171‐220 kg) were included in this study (Table 1). The study was authorized by the Utrecht University Animal Experiments Committee and the Central Committee for Animal Experiments (AVD108002015307).

**Table 1:** Overview of subject characteristics, grooved side, and groove types per joint

Nr	Age at start of study, y	Weight at start of study, kg	Grooved joint	Control joint	Blunt‐grooved joint	Sharp‐grooved joint
1	6	209	Right	Left	Radiocarpal	Middle carpal
2	4	220	Left	Right	Middle carpal	Radiocarpal
3	13	171	Right	Left	Middle carpal	Radiocarpal
4	6	212	Right	Left	Middle carpal	Radiocarpal

### 2.2 Surgical procedure

The grooves were induced in a randomly chosen front limb through an arthrotomy at two locations: the radial facet of the third carpal bone (in the middle carpal joint), and the dorsoproximal surface of the intermediate carpal bone (in the radiocarpal joint). Blunt and sharp grooves were randomly assigned to either one of the joints (Table 1). Blunt grooves were induced with a hooked arthroscopic probe with a sharpened tip; sharp grooves were made with a surgical blade such that incisions were of equal depth and could not exceed 400 μm. The contralateral joints were sham‐operated and used as controls. The ponies were subjected to an exercise program for 8 weeks, starting 3‐weeks post‐surgery.^17^ The ponies were euthanized in week 39, after which the carpal joints were harvested and stored at −20°C.

### 2.3 MRI experiments

MRI experiments were carried out on a 7.0 Tesla whole‐body scanner (Achieva; Philips Healthcare, Best, Netherlands) with a 32‐channel receive head coil (Nova Medical, Wilmington, MA). The carpal joints were placed in the head coil with the dorsal side upwards parallel to the Z‐direction of the magnetic field, such that the articular cartilage surfaces were located orthogonally toward the Z‐direction of the magnetic field.

T2* mapping was carried out by means of a multi‐echo gradient echo (ME‐GRE) sequence with the following readout parameters: three‐dimensional GRE, SENSE factor of 1.5 (AP), TR/TE/ΔTE/FA = 48/4.5/7.3 ms/16°, ProSet fat suppression, field of view = 120 × 120 × 70 mm^3^, resolution = 0.3 × 0.3 × 0.3 mm^3^ with a total acquisition time of 17 minutes and 53 seconds. Four echo times were used: 4.5, 12, 19, and 27 ms, which were optimized based on the expected T2* relaxation time of 15 to 20 ms.

### 2.4 Polarized light microscopy

Osteochondral samples of the whole‐joint surface, including the grooved sites, the kissing sites, and the contralateral controls, were fixed in formalin and decalcified for histology.^17^ Sections of 5 μm, perpendicular to the articular surface and grooves, were stained with Picrosirius Red staining. Stained sections were examined using a light microscope (Olympus BX51) by polarized light microscopy for visualization of collagen fibril orientation.

### 2.5 Image analysis

**Figure 1:**
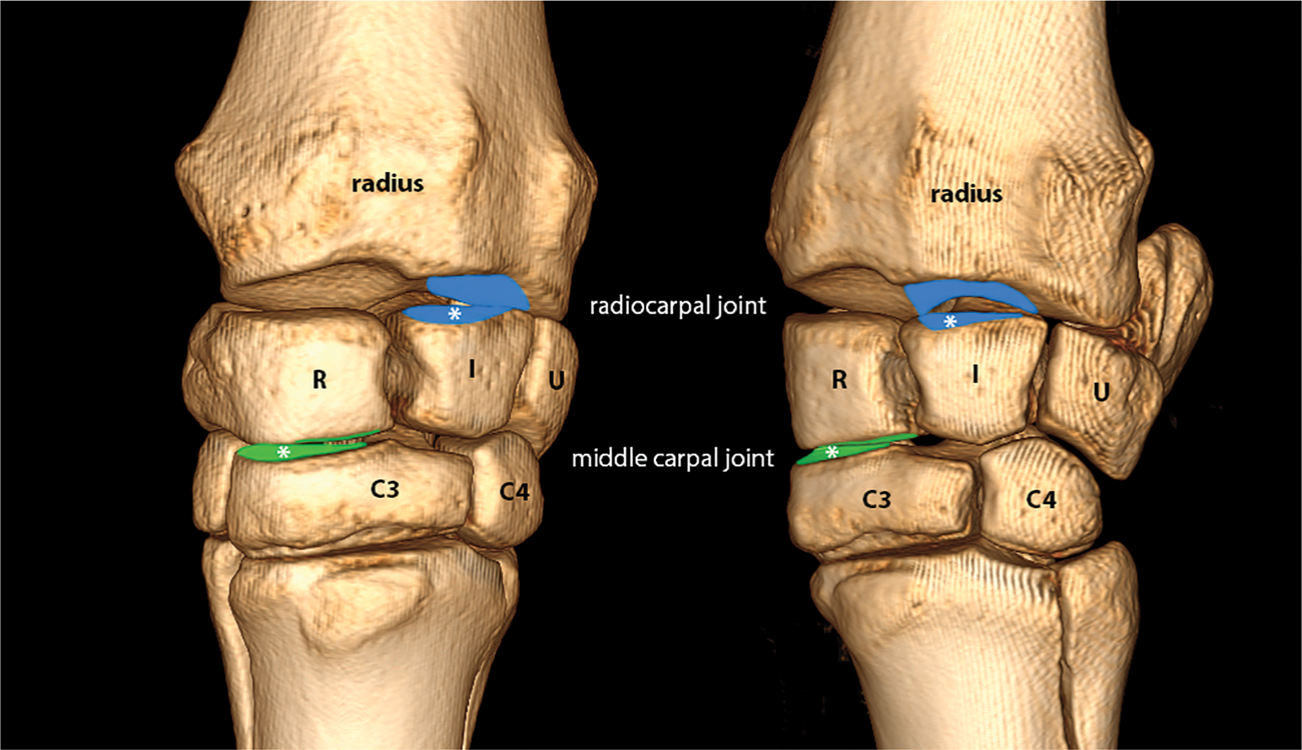
Dorsomedial‐palmarolateral oblique view (left pane) and dorsolateral‐palmaromedial oblique view (right pane) of a left carpus. The following volumes of interests are used in the analysis: distal surface of the radius, dorsoproximal surface of the intermediate carpal bone (I) (blue), distal surface of the radial carpal bone (R), radial facet of the proximal surface of the third carpal bone (C3) (green). *Grooved surfaces [Color figure can be viewed at wileyonlinelibrary.com]

Volumes of interest (VOI) were drawn using ITK‐SNAP (version 3.4.0) in the radiocarpal joint (distal surface of the radius and the dorsoproximal surface of the intermediate carpal bone) and the middle carpal joint (distal surface of the radial carpal bone and radial facet of the third carpal bone). These locations included the grooved and kissing sites and their contralateral controls. This resulted in four VOIs per joint (Figure 1). These VOIs were saved as NIFTI files and transferred to MATLAB (R2017b; The MathWorks, Natick, MA) to carry out the analysis. Fitting an exponential T2* decay function was carried out by a nonlinear least‐squares algorithm.^18^ The average T2* values per VOI were reported and the median T2* values were reported per sample group.

## 3 RESULTS

**Figure 2:**
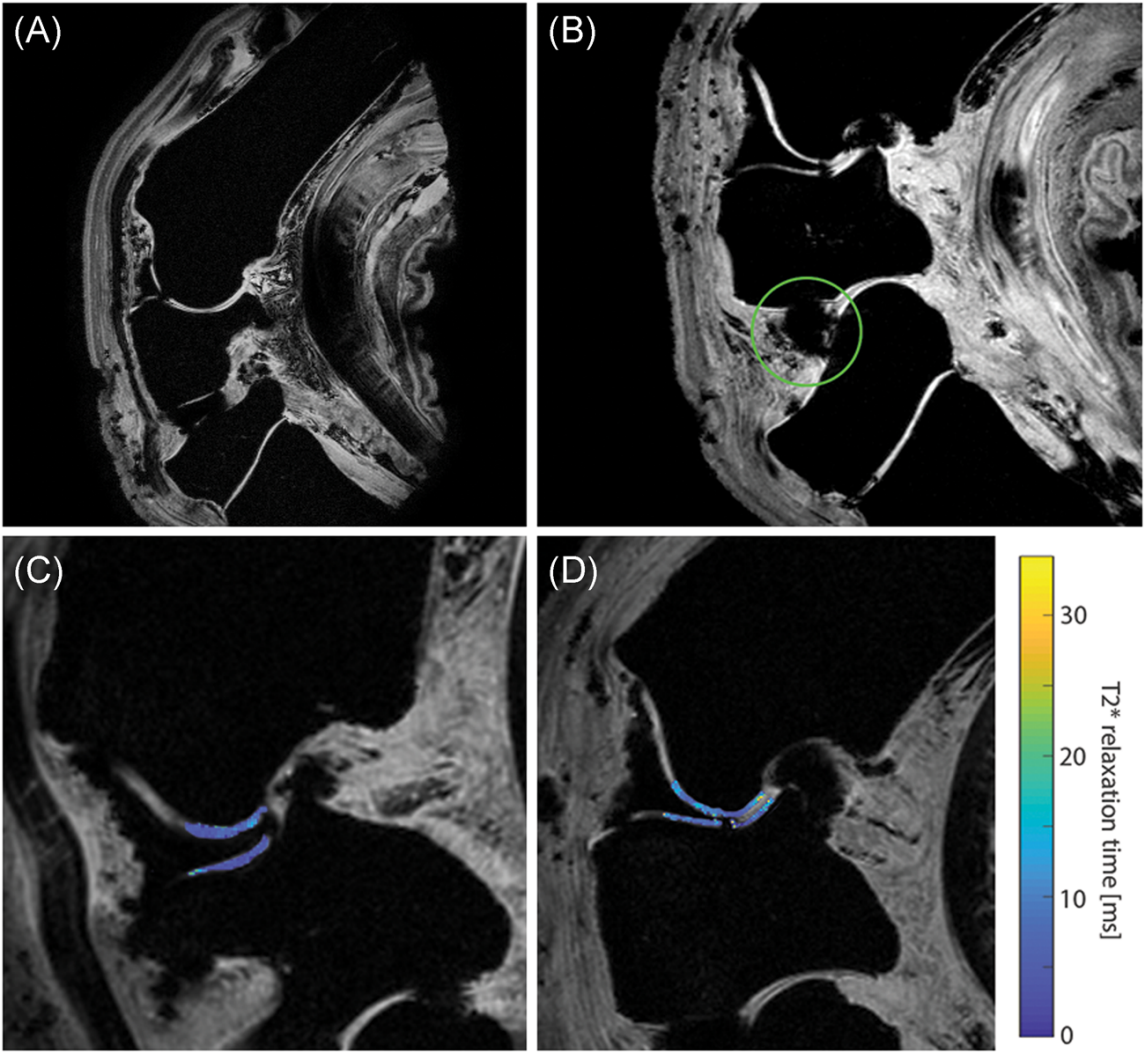
A, Healthy, nongrooved carpus. B, A sagittal slice of the carpus of pony 2, highlighting the air bubble in the intercarpal joint (green circle). C,D, T2* maps showing the radiocarpal joint with sharp‐grooved cartilage (C) and blunt‐grooved cartilage (D, where the cartilage defect due to the groove can be appreciated) [Color figure can be viewed at wileyonlinelibrary.com]

From the 32 sites available (four ponies, two legs/pony, four VOI/leg), a total of 30 sites were included for VOI analysis. Excellent high‐resolution T2* imaging could be performed showing T2* contrast as shown in Figure 2A, with T2* maps of sharp‐grooved and blunt‐grooved cartilage (Figure 2C,D). The left middle carpal joint of pony 2 contained a large air bubble (Figure 2B) caused by the surgery that was performed upon euthanasia (out of the scope of this paper). Therefore, these two sites in this joint were excluded from the analysis.

**Figure 3:**
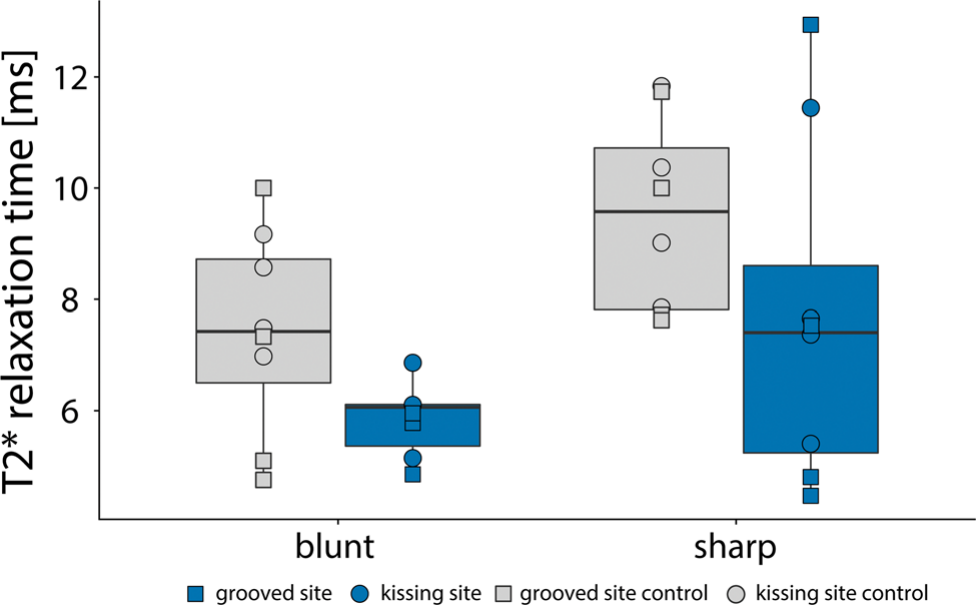
T2* relaxation times in volumes of interests with blunt grooves and sharp grooves compared with contralateral control sites. The grooved sites are shown in blue and control sites in gray [Color figure can be viewed at wileyonlinelibrary.com]

**Figure 4:**
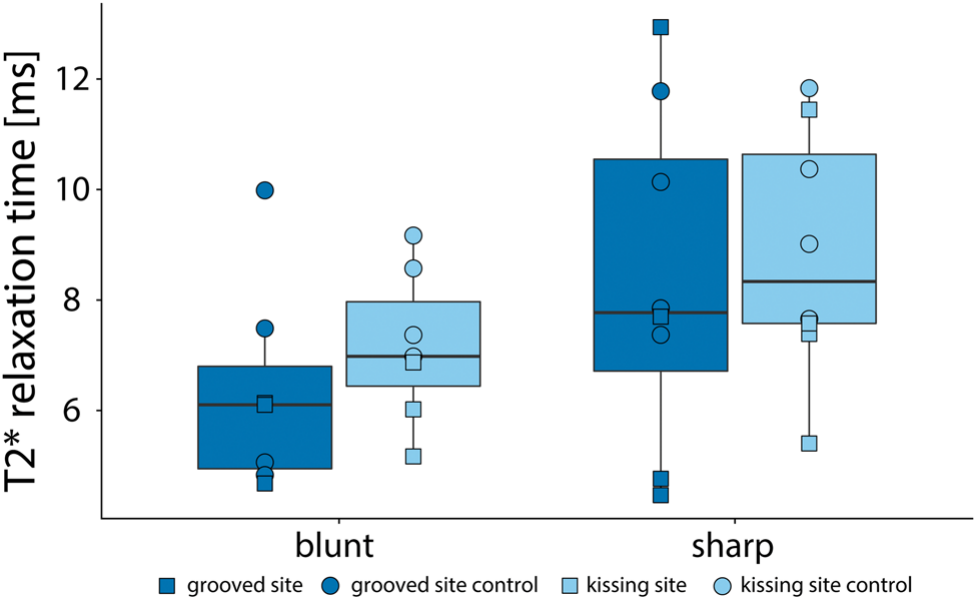
T2* relaxation times in volumes of interests with blunt grooves and sharp grooves compared with their kissing sites. The grooved sites are shown in dark blue and the kissing sites in light blue [Color figure can be viewed at wileyonlinelibrary.com]

The median T2* relaxation time of the sample group of grooved sites was lower (6.06 ms) compared to their contralateral (7.43 ms) counterparts in the blunt‐grooved sample group and similarly in the sharp‐grooved group (7.41 and 9.58 ms, respectively), as shown in Figure 3. Additionally, the median T2* relaxation time of the grooved sites was lower (6.11 ms) as compared to their opposite kissing sites (7.17 ms). Similar patterns were observed in the sharp‐grooved sample group (7.77 and 8.34 ms, respectively) (Figure 4). No substantial differences have been observed in T2* relaxation time between groove types. Median T2* relaxation times have been summarized in Table 2.

**Table 2:** Median T2* relaxation times

	Blunt‐grooved sample group		Sharp‐grooved sample group	
Between joints^a^	Grooved joint	Control joint	Grooved joint	Control joint
	6.06	7.43	7.41	9.58
Within joints^b^	Grooved site	Kissing site	Grooved site	Kissing site
	6.11	7.17	7.77	8.34

^a^ Medians based on grooved and kissing surfaces (Figure 3).

^b^ Medians based on grooved and control sides (Figure 4).

**Figure 5:**
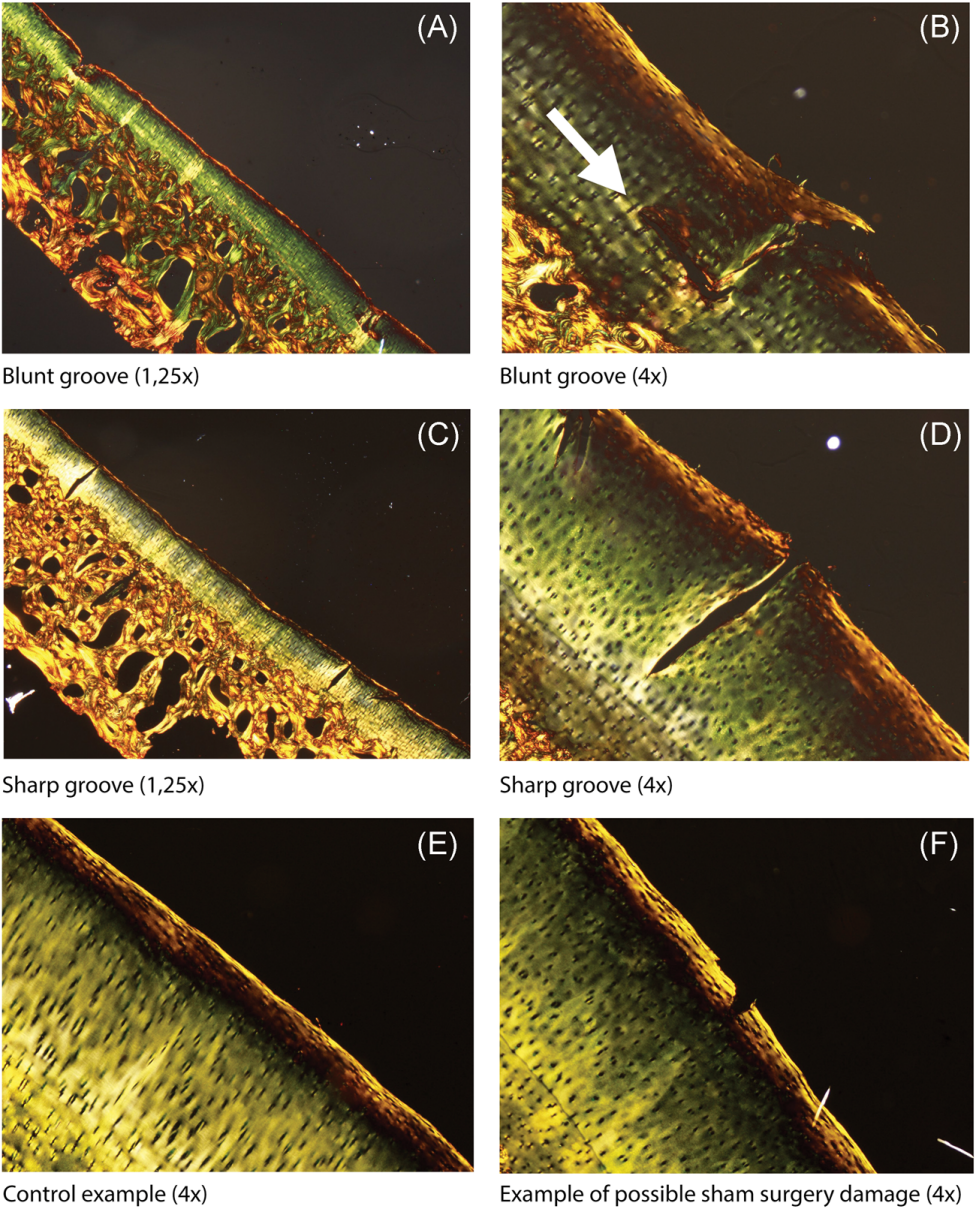
Picrosirius Red‐stained sections analyzed under polarized light. Blunt grooves are shown in (A) (×1.25 magnification) and (B) (×4 magnification). The white arrow points toward a slight disruption of normal collagen orientation. Sharp grooves are shown in (C) (×1.25 magnification) and (D) (×4 magnification). E, An example of a control site. F, An example of possible damage due to sham surgery in a control site [Color figure can be viewed at wileyonlinelibrary.com]

When Picrosirius Red‐stained sections were analyzed under polarized light, typical collagen fiber structures could be recognized in control sites, that is, parallel‐oriented fibers in the superficial layer (showing bright yellow), followed by a zone with low birefringence, first containing obliquely oriented and then perpendicular‐oriented fibers. This collagen structure did not seem to be affected in the cartilage tissue in between grooves. Only in cartilage with blunt lesions, higher birefringence (bright yellow) and slight disruption of normal orientation were observed in all blunt lesions directly around the grooves of which an example is shown in Figure 5. The PLM images nicely showed the relatively unharmed control sites, with one example showing mild damage in the superficial layers of the cartilage. These could be due to the sham surgery performed on the contralateral control sites.

## 4 DISCUSSION

This explorative study investigated T2* relaxation times in equine articular cartilage 39 weeks after application of two types of artificially created grooves (ie, blunt grooves and sharp grooves randomized over the radiocarpal and middle carpal joint), followed by an 8‐week exercise program. The median T2* relaxation time of the sample group of grooved sites was lower compared to their contralateral control sites. Additionally, the median T2* relaxation time of the sample group of grooved sites was lower in relation to their opposite kissing sites.

While a number of analyses are described in more detail in the thesis of te Moller,^17^ the most important ones relating to this work are discussed here. Histology slides were stained with Safranin‐O/Fast‐green to enable scoring using the OARSI histopathological guidelines, showing significantly higher OARSI scores in blunt‐ and sharp‐grooved sections compared to their contralateral control sites. This implies that there was damage in the cartilage, which could be measured with T2* mapping. Additionally, blunt‐grooved sections showed higher OARSI grades than sharp‐grooved sections.^17^ Blunt grooves showed to have an impact on the kissing site, in most samples an imprint was seen opposite to the groove. These two findings possibly explain the generally lower T2* relaxation times in the sample group with blunt‐grooved cartilage. Another explanation could be that the superficial zones are damaged or partly disappeared, such that the average T2* values within the VOI mostly reflect the middle and deep zone of the cartilage. Several studies have shown a tendency to a decrease in T2* relaxation times from superficial to the deep zone.^4^, ^19^ Hesper et al^7^ found that T2* relaxation times were lower in damaged cartilage, as evaluated by arthroscopy. T2* relaxation has also shown to decrease in OA patients when compared with healthy controls.^18^ These results confirm that T2* mapping can be used as a measurement for damaged cartilage.

A limitation of this work is that due to the randomization, grooved sides and groove types were not evenly distributed (ie, three right and one left front limb(s) grooved; three sharp‐grooved radiocarpal joints, one blunt‐grooved radiocarpal joint, and vice versa for the middle carpal joint). This relates to the differences in T2* relaxation time between blunt grooves and sharp grooves which are present, but the differences between the joint types (radiocarpal vs middle carpal) have to be taken into account. The underlying limitation of this is the small sample size within this study—we included four ponies, with eight legs, divided into four groups (being contralateral control blunt, blunt‐grooved, contralateral control sharp, and sharp‐grooved).

One of the limitations of T2* mapping, in general, is that T2* mapping is prone to magic angle artifacts. By placing the samples consequently in the same way in the scanner (dorsal side upwards which caused the cartilage surfaces to be orthogonally oriented towards the magnetic field) we tried to minimize the influence of magic‐angle effects. Cartilage T2 and, therefore, also, T2* was shown to approximately follow the angular dependence of the nuclear dipole‐dipole interaction, which has its maximum at 55°.^20^ This effect is even more pronounced in highly ordered collagenous structures such as the meniscus.^21^ Within this work we specifically chose to implement T2* mapping instead of T2 mapping because it can be implemented with a higher spatial resolution and can be acquired faster, providing more specific information on local tissue disruption that causes steep transitions between tissues of different magnetic susceptibilities.^22^ Nevertheless, other MR methods, less sensitive to magic‐angle artifacts, could have been implemented. T1rho imaging has also been shown to be prone to magic angle effects, depending on which sequence is used.^23^, ^24^ Diffusion tensor imaging (DTI) would also have been an excellent alternative to gain insight in the directionality of the collagen fibril network. The potential of DTI imaging in articular cartilage has been shown ex vivo, where a high spatial resolution of 45 µm was shown in the rat knee.^25^ In vivo work on 7T MRI has shown that DTI can possibly show differences between layers in articular cartilage.^26^

Another limitation of the T2* mapping is the number of echo times, which could be increased for a clinical T2* mapping protocol (four in this work, where at least six would be beneficial for the fitting—although increasing the acquisition time in that case) and the actually chosen echo times can be optimized further. Since the end goal is an imaging protocol which can be used as a biomarker in human cartilage, the comparison is made with human ankle cartilage because of the similar cartilage thickness and load distribution. T2* relaxation values in the human ankle are relatively low, ranging from 11.8 to 20.5 ms.^11^, ^27^ Given these values, we chose four echo times in the range of 4.5 to 27 ms, because of the expected T2* relaxation times reported in the literature. In retrospect, more echo times in the lower range (below 10 ms) would have benefited our results. Additionally, the number of echo times chosen in this work does not allow for bi‐exponential fitting, as is sometimes employed in the analysis of T2* mapping in articular cartilage.

The acquisition time of the T2* mapping protocol used was long due to the high spatial resolution, which has to be taken into account for clinical implementation. The scan time was long because the head coil was used with a corresponding SAR model, which is too conservative for scanning of extremities. With our knee coil, we could increase the SAR (IEC limit for extremities 40 W/kg local SAR instead of 10 W/kg for the head) and, therefore, decrease the acquisition time. Additionally, the SENSE factor can be increased by using receive arrays close to the volume of interest, even decreasing the acquisition time further.

In conclusion, this work shows that T2* relaxation times are different in healthy cartilage vs (early) damaged cartilage as induced by an equine groove model. Additionally, the median T2* relaxation times are different in kissing lesions vs the grooved sites.

## AUTHOR CONTRIBUTIONS

Conception and design: SB and NtM. Analysis and interpretation of the data: SB, NtM, and MF. Drafting of the article: SB and NtM. Critical revision of the article for important intellectual content: SB, NtM, MF, HB, RvW, KI, and DK. Final approval of the article: SB, NtM, MF, HB, RvW, KI, and DK. Provision of study materials or patients: SB and NtM. Statistical expertise: SB and NtM. Obtaining of funding (administrative, technical, or logistic support): SB and NtM, Collection and assembly of data: SB and NtM.
